# Selection of Reference Genes for Quantitative Real-Time PCR Normalization in *Panax ginseng* at Different Stages of Growth and in Different Organs

**DOI:** 10.1371/journal.pone.0112177

**Published:** 2014-11-13

**Authors:** Jing Liu, Qun Wang, Minying Sun, Linlin Zhu, Michael Yang, Yu Zhao

**Affiliations:** 1 Traditional Chinese Medicine and Biotechnology Research and Development Center, Changchun University of Traditional Chinese Medicine, Changchun, Jilin, People's Republic of China; 2 College of Dental Medicine, Columbia University, New York, United States of America; Northwestern University, United States of America

## Abstract

Quantitative real-time reverse transcription PCR (qRT-PCR) has become a widely used method for gene expression analysis; however, its data interpretation largely depends on the stability of reference genes. The transcriptomics of *Panax ginseng*, one of the most popular and traditional ingredients used in Chinese medicines, is increasingly being studied. Furthermore, it is vital to establish a series of reliable reference genes when qRT-PCR is used to assess the gene expression profile of ginseng. In this study, we screened out candidate reference genes for ginseng using gene expression data generated by a high-throughput sequencing platform. Based on the statistical tests, 20 reference genes (10 traditional housekeeping genes and 10 novel genes) were selected. These genes were tested for the normalization of expression levels in five growth stages and three distinct plant organs of ginseng by qPCR. These genes were subsequently ranked and compared according to the stability of their expressions using geNorm, NormFinder, and BestKeeper computational programs. Although the best reference genes were found to vary across different samples, CYP and EF-1α were the most stable genes amongst all samples. GAPDH/30S RPS20, CYP/60S RPL13 and CYP/QCR were the optimum pair of reference genes in the roots, stems, and leaves. CYP/60S RPL13, CYP/eIF-5A, aTUB/V-ATP, eIF-5A/SAR1, and aTUB/pol IIa were the most stably expressed combinations in each of the five developmental stages. Our study serves as a foundation for developing an accurate method of qRT-PCR and will benefit future studies on gene expression profiles of *Panax Ginseng*.

## Introduction

Ginseng (*Panax ginseng C.A. Meyer*) is a perennial herb and is well-known for its adaptogenic and restorative properties. It has been widely used in traditional Chinese medicine and Western herbal medicine [1], [2]. Ginseng root, the most commonly used part of the plant, contains ginsenosides that are major bioactive constituents with complex and multiple pharmacological effects [3], [4]. Ginseng leaf-stem extract also contains numerous important bioactive components [5], [6]. A recent report demonstrated that American ginseng leaf contains similar pharmacologically active ingredients in higher quantity than found in ginseng root [7]. Research has shown that ginseng leaf-stem may as well be a valuable source of ginsenosides as ginseng root [8].

From germination to withering, the stages of growth of ginseng can be generally classified into the leaf-expansion period (LP), the flowering stage (FS), the green fruit stage (GFS), the red fruit stage (RFS), the root growing after fruit stage (RGS), and the withering stage [9]. In recent years, the research focus has expanded considerably towards elucidating the gene expression of ginseng at different developmental stages. Various researchers have highlighted the genetic aspects of ginseng, including the marker gene identification or authentication, genes that confer resistance to environmental and biological stresses, the regulatory factors of its growth and development, and key enzymes involved in the ginsenoside biosynthetic pathway [7], [8], [10]–[15].

qRT-PCR has been widely used as a powerful technique to quantify the expression levels of transcripts. The accuracy of qRT-PCR largely depends on the stability of the reference gene(s) applied to data normalization [16]. A series of presumably stable expressed genes have been used as internal references. Some of the best known and most frequently used reference transcripts, often referred to as housekeeping genes [17], include actin (ACT), tubulin (TUB), glyceraldehyde-3-phosphate dehydrogenase (GAPDH), polyubiquitin (UBQ), and translational initiation factor (eIF). These have been extensively used as reference genes in different organisms because of their stable and uniform expression patterns [18]. However, these references have shown a significant variance when tested across species and under a broad range of experimental tests [19]. Failure to use suitable reference genes may deflect gene expression profiles and lead to misguiding results [16]. So far, there have been no reports on the use of such genes in ginseng. Therefore, it is essential to determine appropriate reference genes in order to undertake genetic engineering studies in ginseng.

Our laboratory has constructed 15 ginseng transcriptome databases (including samples of three organs in five growth stages) using high-throughput sequencing technology. These databases provide more than 73,000 genetic data containing the gene sequences, gene expression levels, gene annotations, and other related information. In the present study, by analyzing the gene annotation process, we aimed to find appropriate reference genes for ginseng. After conducting a comprehensive literature search, the gene expression levels of ten commonly used reference genes [19]–[28] and ten novel expression stable genes were evaluated to select the best candidate reference genes. This selection was based on the statistical tests involving RPKM values at different growth stages and in different organs. In addition, the expressions of 20 candidate genes were measured by qRT-PCR, and the expression stability of each gene was further measured using quantitative software applications, such as geNorm, NormFinder, and BestKeeper. This study provides greater insights into the optimal control genes involving different growth stages and various organs of *P. ginseng*, and will significantly contribute to the development of ginseng transcriptomics.

## Materials and Methods

### Ethics Statement

No specific permissions were required for the locations used or activities undertaken in the present study. The samples of *Panax ginseng C.A Meyer* were originally collected from Fu-song County (longitude: 127.28, latitude: 42.33), Jilin province, China. No endangered or protected species were involved in the field studies.

### Plant material

Five stages of *P. ginseng* were harvested from Fu-song County, Jilin province, China. 5-year-old ginseng plants were used for library construction. After cleaning with distilled water, the main roots, stems, and leaves were minced into small pieces, and immediately frozen in liquid nitrogen.

### Total RNA samples

Total RNA was isolated using TRIzol reagent (Invitrogen) according to the manufacturer's instructions. Quality of RNA was ascertained by measuring absorbance at 260 nm using the BioSpec-nano Spectrophotometer and through 1% ethidium bromide (EtBr)-stained agarose gel electrophoresis. The total RNA integrity [29] was further tested using the 2100 Bioanalyzer (Agilent Technologies).

### cDNA library construction, sequencing, assembly, and gene expression analyses

The samples, processed according to the Illumina kit instructions, were prepared for the transcriptome analysis. Protocols for the cDNA library construction, sequencing, assembly, and gene expression level analysis have been previously described by Baojin Yao [30]. Based on the RPKM values, the estimated gene expression was used directly for comparing the differences in gene expressions between samples. Distinct sequences were used for the BLAST search and annotated against the NCBI nr database using an E-value cut-off of 10^−5^ [31].

Using the Illumina sequencing platform, we generated more than 39 million high-quality sequencing reads for each sample. After clustering via the TGICL software, more than 80,000 unigenes were produced in every database. Unigene sequences were aligned by BLASTX to four common protein databases (Nr, Swiss-Prot, KEGG, and COG; e-value <0.00001). Simultaneously, we obtained the highest sequence similarities Unigenes along with their protein functional annotations.

### Selection of candidate reference genes for normalization

On analyzing the existing databases, ten commonly used housekeeping genes were selected as endogenous control genes. Based on the calculated statistical values of the coefficient of variation (CV  =  SD/Mean) and the maximum fold change (MFC  =  Max_(RPKM)_/Min_(RPKM)_) [32], we obtained ten novel reference genes from the 15 databases.

In total, 20 candidate reference genes were selected, including 10 housekeeper reference genes (ACT1, GAPDH, UBQ, 18SrRNA, eIF-5A, aTUB, bTUB, CYP, F-box, and EF-1α) and 10 novel reference genes (CDP, 6-PG, 30S RPS20, 60S RPL13, V-ATP, pol IIa, ARF, QCR, SAR1, and TCTP).

### Primer design and validation

Based on the sequences obtained from high-quality cDNA sequencing, primers were designed using primer 5.0 software. The specificity of the primers was confirmed by BLAST searches.

In order to examine the target specificity of primers, reverse transcription PCR was employed. With 500 ng of total RNA (each from five stages) as the template, a thermal cycling profile was conducted according to the following protocol: 30°C for 10 min, 50°C for 30 min, 95°C for 5 min, 5°C for 5 min; 30 cycles at 94°C for 30 s, 60°C for 30 s, 72°C for 1 min. The products were visualized by 2% agarose gel electrophoresis along with the DL1000 DNA marker.

### Quantitative Real-Time PCR

The test of transcript variability among the fifteen samples (three organs and five stages) was carried out using qRT-PCR reactions for mRNA. These reactions were performed in triplicate using the MxPro 4.1 system assays and the One Step SYBR PrimeScript PLUS RT-PCR kit (TaKaRa, TaKaRa code: DRR096A), including minus reverse transcription (RT) controls to assess the genomic DNA and non-template controls, thereby ensuring a lack of background signal in the assay. The final volume of the RT reaction was 25 µl, which consisted of 12.5 µl 2×One Step SYBR RT-PCR Buffer, 1.5 µl TaKaRa Ex Taq HS Mix, 0.5 µl PrimeScript PLUS RTase Mix, 10 µM PCR Forward Primer, 10 µM PCR Reverse Primer, 40 ng total RNA, and 6.5 µl RNase-free H_2_O. The reactions were incubated in thin-wall polypropylene 8-tube strips using MxPro 4.1. The PCR cycling conditions were as follows: 42°C for 5 min, 95°C for 10 sec, followed by 40 cycles of 95°C for 5 sec and 60°C for 30 sec. Finally, the steps, 95°C for 15 sec, 60°C for 30 sec, and 95°C for 15 sec were carried out for dissociation. Data were collected during each cycle at the 60°C extension step.

### Analysis of stability of candidate reference genes

The variation among 20 reference genes was determined by cycle threshold (Ct) using the MxPro 4.1 software, following the manufacturer's instructions. Generally, the Ct value of every single reaction and the mean efficiency of each amplicon were used to calculate their relative expression levels [17]. To compare the stability of the 20 candidate reference genes, three Visual Basic Applications (VBA) for Microsoft Excel – geNorm (http://medgen.ugent.be/~jvdesomp/genorm/), NormFinder (http://www.mdl.dk/publicationsnormfinder.html), and BestKeeper (http://www.gene-quantification. de/bestkeeper.html) were used. The Ct values of the candidate reference genes were divided into nine sets of samples for further analysis, which included the total set (all data set), roots, stems, leaves, LP, FS, GFS, RFS, and RGS.

## Results

### Screening of the candidate reference genes

**Figure 1:**
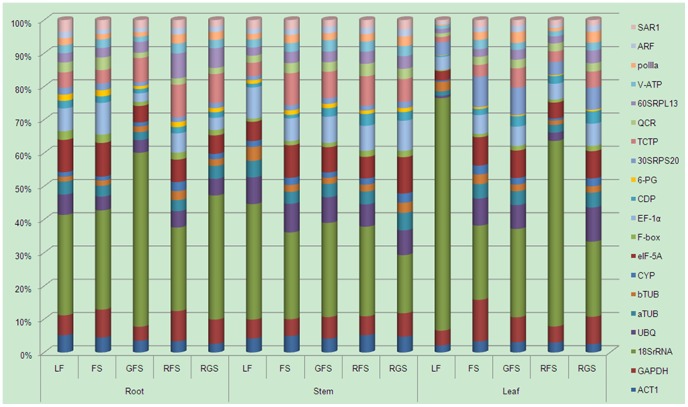
RPKM value distribution of 20 candidate reference genes. LP, leaf-expansion period; FS, the flower stage; GFS, the green fruit stage; RFS, the red fruit stages; RGS, the root growing after fruit stage.

In the present study, we screened ten housekeeping genes (ACT1, GAPDH, 18SrRNA, UBQ, aTUB, bTUB, CYP, eIF-5A, F-box, and EF-1α). Besides 18S rRNA, the RPKM value distribution of the remaining nine housekeeping genes was in the range of 90–500. According to this observation, the RPKM value selection range of the candidate reference genes was expanded to 50–500. To evaluate the gene expression volatility, we examined the variability in PRKM values among the 15 databases. The CV and MFC values of the ten traditional housekeeping genes were calculated in one organ during the five stages of growth or in the three vegetative organs at one growth stage (Table 1-a). The CV values of the housekeeping genes were found to vary from 3.06% to 88.21%, while the MFC values ranged from 1.06 to 8.76. In order to screen more stable reference genes, we set the threshold values for CV to <20% and MFC to <1.5. Additionally, ten novel genes (CDP, 6-PG, 30S RPS20, 60S RPL13, V-ATP, pol IIa, ARF, QCR, SAR1, and TCTP) were screened as candidate reference genes (Table 1-b). Screening of the potential reference genes was based on the statistical tests (CV and MFC), which reflected the RPKM values of stably and moderately or highly expressed genes among all the databases. RPKM value expression abundance ratios are presented in Figure 1. To determine the distribution of transcript populations of 20 candidate reference genes in three vegetative organs of ginseng during the five stages of growth, the quantity of transcript for each gene was estimated as a ratio relative to the sum of the 20 transcript populations. The results clearly revealed a fluctuation in the relative magnitude of RPKM values and the ratios, thus indicating that all of the 20 genes did not exhibit stable expression patterns. A summary of the sequence information for the 20 ginseng candidate reference genes is presented in Table 2.

**Table 1:** Variability of the candidate reference genes in the different samples.

	Root	Stem	Leaf	LP	FS	GFS	RFS	RGS
**Gene**	ACT1	ACT1	ACT1	ACT1	ACT1	ACT1	ACT1	ACT1
**Mean**	280.27	265.46	156.12	283.56	249.51	219.06	229.38	188.24
**CV(%)**	17.25	13.13	36.75	12.10	34.39	50.76	21.21	40.66
**MFC**	1.56	1.39	2.39	1.26	2.01	3.03	1.53	2.43
**Gene**	GAPDH	GAPDH	GAPDH	GAPDH	GAPDH	GAPDH	GAPDH	GAPDH
**Mean**	517.62	392.02	392.02	422.62	478.44	335.02	416.59	434.14
**CV(%)**	26.50	14.19	34.86	18.40	31.78	19.14	53.80	33.16
**MFC**	1.91	1.40	2.11	1.44	1.88	1.48	2.38	1.80
**Gene**	18S rRNA	18S rRNA	18S rRNA	18S rRNA	18S rRNA	18SrRNA	18S rRNA	18S rRNA
**Mean**	2726.48	1563.01	2829.04	4088.88	1527.74	2449.55	2167.32	1630.74
**CV(%)**	48.92	36.53	88.21	83.35	32.45	88.20	45.18	75.91
**MFC**	2.77	2.70	8.76	4.49	1.97	5.18	2.37	3.35
**Gene**	UBQ	UBQ	UBQ	UBQ	UBQ	UBQ	UBQ	UBQ
**Mean**	354.75	446.27	254.84	340.27	388.34	338.54	285.81	406.81
**CV(%)**	13.56	21.62	55.54	72.62	31.01	21.85	42.02	3.06
**MFC**	1.48	1.67	4.99	6.91	1.86	1.56	2.48	1.06
**Gene**	bTUB	bTUB	bTUB	bTUB	bTUB	bTUB	bTUB	bTUB
**Mean**	142.86	155.96	135.11	234.19	119.91	109.24	126.68	133.19
**CV(%)**	33.36	53.08	79.35	56.06	10.99	42.01	52.14	36.77
**MFC**	2.41	3.09	4.51	3.87	1.25	2.25	2.70	2.14
**Gene**	aTUB	aTUB	aTUB	aTUB	aTUB	aTUB	aTUB	aTUB
**Mean**	248.24	250.74	162.62	245.46	210.32	194.88	193.14	258.86
**CV(%)**	16.39	26.37	15.59	42.05	5.54	20.96	28.60	27.35
**MFC**	1.46	1.79	1.32	2.36	1.11	1.53	1.81	1.76
**Gene**	CYP	CYP	CYP	CYP	CYP	CYP	CYP	CYP
**Mean**	121.36	116.79	75.50	87.57	109.78	88.71	111.44	125.26
**CV(%)**	41.12	22.39	45.42	44.66	24.99	23.95	74.70	20.54
**MFC**	2.57	1.54	3.29	2.52	1.65	1.59	37.10	1.52
**Gene**	eIF-5A	eIF-5A	eIF-5A	eIF-5A	eIF-5A	eIF-5A	eIF-5A	eIF-5A
**Mean**	536.09	460.75	324.73	438.19	552.48	386.54	371.38	454.03
**CV(%)**	17.46	24.78	12.60	28.52	27.24	22.44	30.90	26.74
**MFC**	1.50	1.77	1.38	1.75	1.76	1.58	1.78	1.73
**Gene**	F-box	F-box	F-box	F-box	F-box	F-box	F-box	F-box
**Mean**	143.55	80.05	48.39	84.49	95.38	75.92	99.56	97.99
**CV(%)**	15.16	18.83	23.15	73.19	66.87	42.88	52.28	34.08
**MFC**	2.50	1.49	1.92	4.59	3.80	1.70	3.02	2.04
**Gene**	EF-1a	EF-1a	EF-1a	EF-1a	EF-1a	EF-1a	EF-1a	EF-1a
**Mean**	403.63	470.07	293.66	509.99	435.84	286.42	364.72	348.64
**CV(%)**	37.91	25.04	31.75	27.82	44.13	37.61	21.18	32.12
**MFC**	2.64	1.74	2.18	1.66	2.47	1.97	1.54	1.77
**Gene**	CDP	V-ATP	TCTP	60SRPL13	V-ATP	pol IIa	CDP	CDP
**Mean**	133.83	163.71	188.49	166.52	151.67	117.42	152.08	142.48
**CV(%)**	5.11	6.05	8.84	5.81	15.47	7.60	16.47	4.53
**MFC**	1.14	1.14	1.28	1.12	1.37	1.16	1.34	1.09
**Gene**	30SRPS20	ARF	QCR	QCR	pol IIa	CDP	QCR	pol IIa
**Mean**	121.44	103.96	112.80	149.79	119.79	123.19	157.29	160.48
**CV(%)**	11.58	6.42	16.32	17.70	19.34	9.77	17.92	19.30
**MFC**	1.31	1.19	1.50	1.43	1.40	1.11	1.36	1.47
**Gene**	SAR1	60SRPL13	60SRPL13	V-ATP	ARF			
**Mean**	195.68	187.47	129.78	143.11	86.04			
**CV(%)**	12.65	8.03	19.65	18.90	19.53			
**MFC**	1.39	1.21	1.49	1.46	1.50			
**Gene**	6-PG	30SRPS20	SAR1					
**Mean**	105.83	88.88	82.35					
**CV(%)**	14.48	10.38	19.23					
**MFC**	1.49	1.34	1.50					
**Gene**	ARF	QCR						
**Mean**	119.60	166.70						
**CV(%)**	17.00	10.92						
**MFC**	1.41	1.24						
**Gene**	V-ATP	pol IIa						
**Mean**	177.53	128.55						
**CV(%)**	19.70	15.33						
**MFC**	1.47	1.39						

Notes: Descriptive statistics of the candidate genes based on the coefficient of variance (CV) and the maximum fold change (MFC). In total, 10 untraditional reference genes were screened, which had the CV less than 20% and MFC less than 1.5. LP, leaf-expansion period; FS, the flower stage; GFS, the green fruit stage; RFS, the red fruit stages; RGS, the root growing after fruit stage.

**Table 2:** *Panax ginseng* candidate reference genes, primers, amplicon characteristics.

Gene Symbol	Gene name	GenBank Accession Number	Primer sequence (5' → 3')	Tm (°C)	Amplicon Length(bp)
ACT1	actin 1	KF699319	TGGCATCACTTTCTACAACG;TTTGTGTCATCTTCTCCCTGTT	55.8;53.9	109
GAPDH	glyceraldehyde-3-phosphate dehydrogenase	KF699323	GAGAAGGAATACACACCTGACC;CAGTAGTCATAAGCCCCTCAAC	57.7; 57.7	124
18SrRNA	18S ribosomal RNA	KF680553	TTCACACCAAGTATCGCATTTC;CCAAGGAAATCAAACTGAACTG	53.9; 55.8	145
UBQ	polyubiquitin	KF680557	AACCAACTGATACCATTGACCG;CTTTTGCTGTTTTGTCATCTCC	55.8; 53.9	120
aTUB	tubulin alpha-1 chain	KF680556	CTCTGTTGTTGGAACGCTTGTC;CTGTGTGCTCAAGAAGGGAATG	57.757.7	144
bTUB	beta-tubulin	KF699320	TGTTGTGAGGAAAGAAGCCGAG;GGAGAAGGGAAGACAGAGAAAG	57.7;57.7	140
eIF-5A	translational initiation factor eIF-5a	KF680554	CGGCACCATCCGTAAGA;AGCAGGGCGTCATCAGTT	54.6;54.9	300
EF-1α	elongation factor 1-alpha	KF699322	ATAAGCCCCTTCGTCTCCC;CCAAAAGTCACAACCATACCG	57.3;55.6	115
CYP	cyclophilin	KF699321	CAGGCAAAGAAAAAGTCAAGTG;AAAGAGACCCATTACAATACGC	53.9;53.9	108
F-box	F-box containing protein	KF680555	GGTTGCTTTCTGTTGCTTATTA;CCCTTTGATTACTTTTCGCCTG	52.1;55.8	236
CDP	coil domain protein	KF574819	TTCCATCCAAGGTAACAAGGTG;ATCCGTTTCTCCACTCTCACAG	55.8;57.7	144
6-PG	Glucose-6-phosphate/phosphate translocator	KF699324	GTGGGCACTTGGATGGAAAACT;CCAATGCTAAATGTCAAGGGAG	57.7;55.8	147
60S RPL13	60S ribosomal protein L13	KF699330	GGGACTGGTAAGGCAGAAAATG;CTGCTGCTCCTCGCTTAGTCTT	57.7;59.5	155
30S RPS20	30S ribosomal protein S20	KF699325	CCCGAATGAAGAAGGTTTTG;GGGCTTGGGAGAAGGTGTAT	53.4;57.4	236
V-ATP	V-type proton ATPase subunit B	KF699328	AAGAGTGCCATTGGTGAGG;CCTTGAGCGACAAACTTCC	55.2;55.2	191
Pol IIa	DNA-directed RNA polymerase IIa	KF699327	TGAGCCGATTGAACCAGAGC;CACCCTCCAACTCAACCATCAC	57.4;59.5	242
ARF	ADP-ribosylation factor	KF699326	TGAGGATGAACTTAGGGATGCT;CCTTCATAAAGTCCCTCACCTG	55.8;57.7	171
QCR	ubiquinol-cytochrome C reductase	KF680558	CCTCGTCCTAAAGTTTGTTCTC;TCACAGTGCTTCCAGGTTCA	55.8;55.4	104
SAR1	Small GTP-binding protein sar1	KF699329	TTCTTCTGGATTGGTTCTATGG;TGTCGGTTGATGCTGAACTAAT	53.9;53.9	149
TCTP	translationally controlled tumor protein	KF680559	TGGGAAGTTGAGGGAAAGTG;AAATGTGTCAACAATGTCAACC	55.4;52.1	138

### Validating the expression levels of candidate reference genes by qRT-PCR

**Figure 2:**
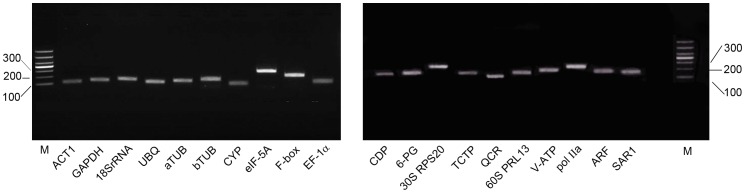
Specificity of primer pairs for RT-qPCR amplification. Agarose gel (2%) electrophoresis showing amplification of a specific PCR product of the expected size for each gene (M:DL1000 DNA Marker).

By reverse transcription PCR, the specificity of the primers used for candidate reference genes was verified. A single band for each gene was revealed through electrophoresis, without primer-dimers or non-specific amplification (Figure 2).

Based on SYBR Green detection, qRT-PCR analysis was employed to evaluate the stability of the expressions of the 20 candidate reference genes in different organs and different developmental stages of *P. ginseng*. The samples were divided into fifteen groups comprising of three organs (roots, stems, and leaves) and five developmental stages. The Ct values of the reference genes of each group were then used to compare the various degrees of expression.

### Statistical data analysis

The gene expression data were analyzed by Ct value, geNorm, NormFinder, and BestKeeper applets to obtain the expression stability of 20 candidate reference genes.

**Figure 3:**
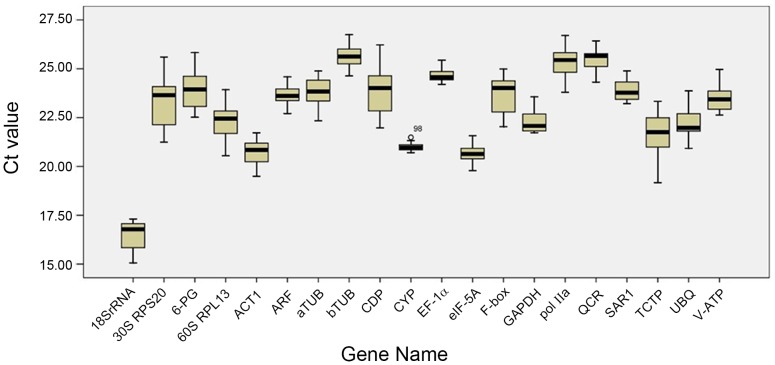
RT-qPCR CT values for the candidate reference genes (n = 3). Expression date displayed as CT values for each reference gene in all ginseng samples. A line across the box is depicted as the median. The box indicates the 25th and 75th percentiles. Whiskers represent the maximum and minimum values.

With a higher gene expression, a smaller Ct value was obtained, and vice versa. Figure 3 shows a relatively broad range of Ct values for all the 20 putative reference genes. The highest Ct value was 26.40 (bTUB), while the lowest was 15.06 (18S rRNA). Ct values of the remaining genes were distributed between 19 and 24. On comparing the Ct values of the 20 candidate reference genes, the expression level of each reference gene was found to differ, with respect to the developmental stage or the organ under study. The expression patterns of the 20 reference genes displayed irregular variation; this may be attributed to change in the level of reference gene expression abundance with the cell type and the developmental stage [33]. Therefore, successful gene expression analysis under different experimental conditions in ginseng requires careful selection of reliable reference genes.

**Figure 4:**
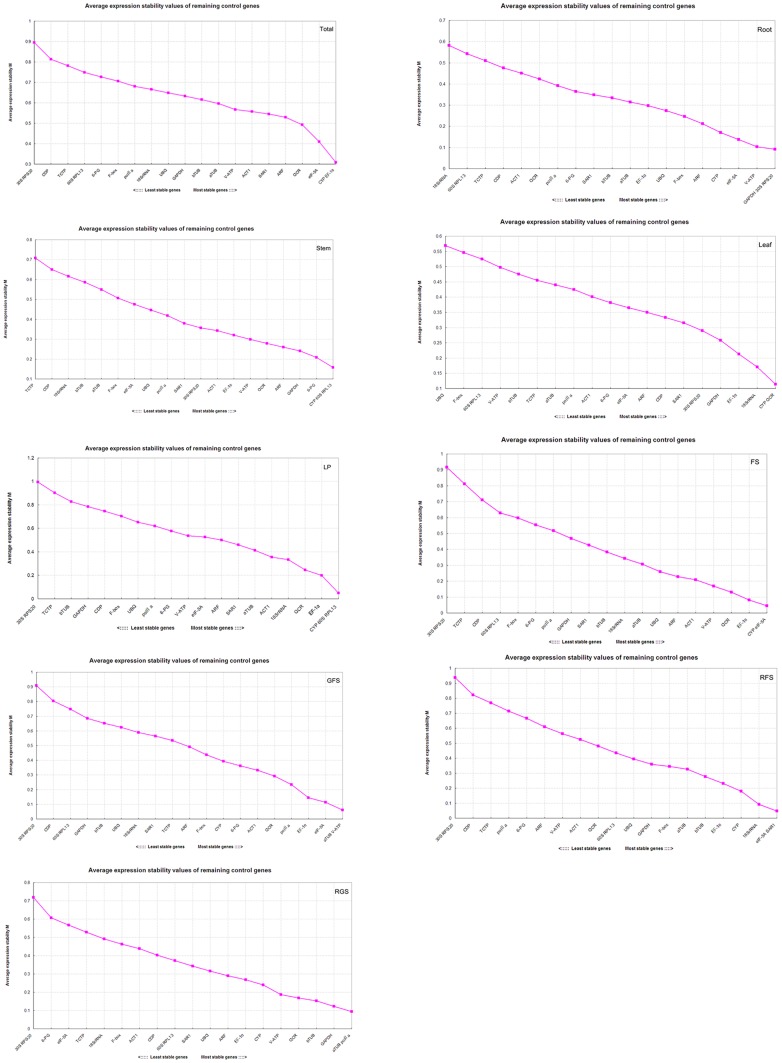
Gene expression stability and ranking of 20 candidate reference genes as caluculated by geNorm. The stability value (M) was determined by assessing the mean pairwise variations of all genes; the least stable gene (the highest M value) was excluded, and the M value was recalculated until the most stable pair was selected.

**Figure 5:**
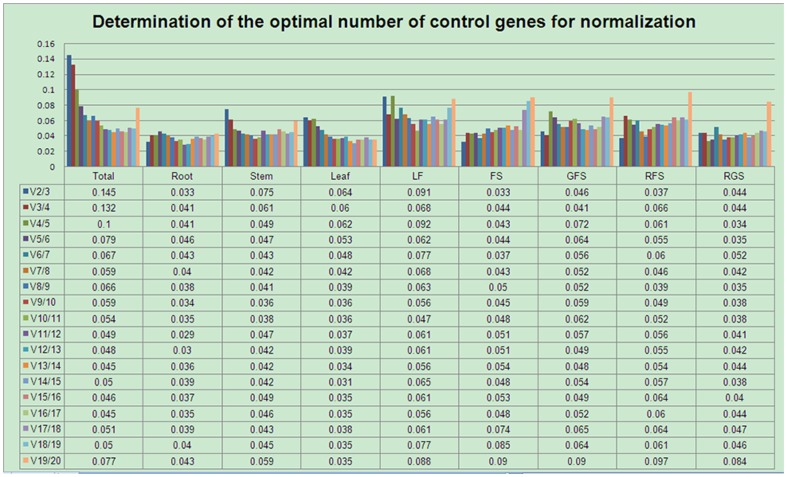
Determination of the optimal number of reference genes required for effective normalization. The geNorm program calculated an NF and used the variable V to determine pairwise variation (Vn/Vn+1) between two sequential NFs (NFn and NFn+1). Additional genes are included when V exceeds the cutoff value, which is typically set at 0.15 but is not always achievable. The number of reference genes is deemed optimal when the lowest possible V value is achieved, at which point it is unnecessary to include additional genes in the normalization strategy.

Based on the expression stability of the genes and the assumption that two ideal reference genes should not vary with each other under different test conditions [34], geNorm ranked the best out of the three data analysis applications used. geNorm computes the average pair-wise variation of a given candidate reference gene with all the other genes and assigns a score of its expression stability (M) to each gene. Stepwise exclusion of genes with the highest M values (indicating the least stable expressions) before recalculation finally reveals the two most stable candidate genes [35]. After calculating the pair-wise variation Vn/n+1, geNorm selects the optimal number of control genes. The cut-off value is usually set to a default value of 0.15 [34]. Gene expression stability and ranking of 20 candidate reference genes, as calculated by geNorm using nine sets of samples, are presented in Figure 4. Analyses of all fifteen samples revealed that the CYP and EF-1α combination showed the lowest M value (0.31), while 30S RPS20 showed the highest M value (0.89). Among the different organs, GAPDH/30S RPS20, CYP/60S RPL13, and CYP/QCR were the most stably expressed gene combinations in roots, stems, and leaves, respectively; while 18S rRNA, UBQ, and TCTP were the least stably expressed. Among the five developmental stages under study, CYP/60S RPL13, CYP/eIF-5A, aTUB/V-ATP, eIF-5A/SAR1, and aTUB/pol IIa were the most stably expressed combination, respectively, and 30S RPS20 was the least stably expressed gene in all the five stages. Based on these observations, CYP was evidently the most stably expressed gene and may be considered as the most suitable reference gene for the analyses of gene expressions in *P. ginseng.* Furthermore, the addition of a third reference gene would not have significantly increased the statistical reliability of this calculation, as V2/3 = 0.033 or V3/4 = 0.041 (in roots) was significantly below the default cut-off value of 0.15 (Figure 5). Although the pair-wise variation for all the samples (V2/3) was estimated as 0.145, it was still less than the limiting value. Hence, our study showed that two reference genes were sufficient to normalize gene expression for all the samples of *P. ginseng*.

The NormFinder algorithm uses a model-based approach to evaluate modifications amongst the reference gene expression levels [36]. Similar to the geNorm method, NormFinder imparts a score of expression stability (M) to each gene, which is negatively correlated with the stability of gene expression [37]. In addition, NormFinder can determine the estimated inter- and intra-group variances [38]. The calculated values generated by NormFinder are shown in Table 3. In the final outcome, CYP, QCR, and aTUB show the most stable expression levels for the total samples, stems, leaves, and the five developmental stages, while 30SRPS20 and TCTP were observed to be less stable. In the roots, GAPDH and V-ATP were the most stably expressed genes with values of 0.013 and 0.110, while 18S rRNA was the least stable. Nevertheless, EF-1α and eIF-5A were found to be in the forefront of the rankings. The results of NormFinder and geNorm were almost consistent.

**Table 3:** Ranking of candidate reference genes in order of their expression stability as calculated by NormFinder software.

Rank	Total	Root	Stem	Leaf	LP	FS	GFS	RFS	RGS
**1**	CYP	GAPDH	QCR	CYP	CYP	aTUB	aTUB	aTUB	QCR
**M value**	0.227	0.013	0.095	0.040	0.114	0.038	0.021	0.013	0.032
**2**	QCR	V-ATP	ARF	QCR	60S RPL13	CYP	V-ATP	F-box	V-ATP
**M value**	0.255	0.110	0.105	0.046	0.146	0.062	0.021	0.013	0.032
**3**	eIF-5A	30SRPS20	EF-1α	18SrRNA	QCR	UBQ	eIF-5A	18SrRNA	aTUB
**M value**	0.268	0.114	0.130	0.075	0.157	0.070	0.062	0.118	0.033
**4**	EF-1α	CYP	30SRPS20	EF-1α	EF-1α	V-ATP	QCR	bTUB	pol IIa
**M value**	0.293	0.148	0.134	0.169	0.243	0.094	0.143	0.165	0.093
**5**	ACT1	6-PG	GAPDH	GAPDH	eIF-5A	eIF-5A	EF-1α	eIF-5A	bTUB
**M value**	0.301	0.158	0.186	0.183	0.282	0.105	0.147	0.195	0.109
**6**	V-ATP	SAR1	6-PG	ARF	V-ATP	EF-1α	pol IIa	SAR1	GAPDH
**M value**	0.306	0.182	0.190	0.191	0.303	0.182	0.204	0.222	0.125
**7**	GAPDH	EF-1α	60SRPL13	SAR1	ARF	18SrRNA	ACT1	GAPDH	SAR1
**M value**	0.342	0.205	0.256	0.210	0.312	0.188	0.243	0.300	0.209
**8**	ARF	eIF-5A	CYP	CDP	18SrRNA	QCR	6-PG	QCR	ARF
**M value**	0.362	0.211	0.258	0.226	0.370	0.273	0.291	0.314	0.237
**9**	UBQ	ARF	ACT1	30SRPS20	SAR1	ACT1	CYP	CYP	60SRPL13
**M value**	0.368	0.216	0.283	0.232	0.406	0.320	0.307	0.339	0.258
**10**	pol IIa	F-box	V-ATP	ACT1	ACT1	ARF	UBQ	ACT1	UBQ
**M value**	0.383	0.224	0.287	0.259	0.408	0.329	0.352	0.351	0.300
**11**	SAR1	bTUB	UBQ	eIF-5A	aTUB	pol IIa	bTUB	EF-1α	CYP
**M value**	0.386	0.259	0.290	0.260	0.467	0.375	0.398	0.368	0.303
**12**	aTUB	aTUB	pol IIa	TCTP	pol IIa	bTUB	F-box	60SRPL13	EF-1α
**M value**	0.405	0.277	0.320	0.301	0.503	0.391	0.441	0.391	0.308
**13**	bTUB	UBQ	eIF-5A	6-PG	GAPDH	GAPDH	GAPDH	V-ATP	CDP
**M value**	0.427	0.344	0.352	0.312	0.525	0.405	0.491	0.405	0.336
**14**	F-box	ACT1	SAR1	aTUB	6-PG	6-PG	ARF	UBQ	ACT1
**M value**	0.443	0.344	0.352	0.336	0.593	0.456	0.534	0.506	0.367
**15**	18SrRNA	QCR	F-box	pol IIa	bTUB	F-box	TCTP	ARF	F-box
**M value**	0.453	0.351	0.412	0.338	0.634	0.509	0.599	0.568	0.371
**16**	60SRPL13	pol IIa	aTUB	bTUB	UBQ	60SRPL13	SAR1	pol IIa	18SrRNA
**M value**	0.476	0.386	0.542	0.418	0.663	0.528	0.609	0.715	0.426
**17**	6-PG	CDP	CDP	F-box	CDP	SAR1	18SrRNA	6-PG	TCTP
**M value**	0.519	0.411	0.552	0.432	0.678	0.547	0.671	0.719	0.550
**18**	CDP	TCTP	18SrRNA	60SRPL13	F-box	CDP	60SRPL13	CDP	eIF-5A
**M value**	0.635	0.467	0.566	0.436	0.695	0.928	0.765	0.780	0.574
**19**	TCTP	60SRPL13	bTUB	V-ATP	TCTP	TCTP	CDP	TCTP	6-PG
**M value**	0.672	0.506	0.568	0.461	0.949	1.166	0.795	0.832	0.665
**20**	30SRPS20	18SrRNA	TCTP	UBQ	30S RPS20	30SRPS20	30SRPS20	30SRPS20	30SRPS20
**M value**	1.070	0.591	0.813	0.484	1.216	1.240	1.242	1.337	1.166

Notes: LP, leaf-expansion period; FS, the flower stage; GFS, the green fruit stage; RFS, the red fruit stages; RGS, the root growing after fruit stage.

The stability of the candidate reference gene expression was also analyzed using BestKeeper, an Excel-based tool. In this analysis, the average Ct value of every single reaction is applied to analyze the stability of each candidate reference gene [25]. Rankings of the candidate reference genes are based on their pair-wise correlation with this index value, which is indicated by the Pearson correlation coefficient (r) [35]. BestKeeper calculates the standard deviation (SD) and the coefficient of variation (CV) based on the Ct values. The most stable reference genes exhibit the lowest CV and SD (CV±SD) [39]. Because the maximum number of genes analyzed by this algorithm is 10 [40], the candidate genes that rank lower in the previous analyses are generally ruled out. The ranking of the genes revealed through BestKeeper analysis is presented in Table 4. These results were mostly consistent with those obtained using geNorm, including the total samples, roots, stems, leaves and LP.

**Table 4:** Ranking of candidate reference genes in order of their expression stability as calculated by BestKeeper software.

Rank	Total	Root	Stem	Leaf	LP	FS	GFS	RFS	RGS
**1**	CYP	GAPDH	CYP	CYP	60SRPS20	ACT1	CYP	CYP	CYP
**CV%±SD**	0.85±0.18	0.37±0.08	0.71±0.15	0.53±0.11	0.41±0.09	0.29±0.06	1.04±0.22	0.02±0.00	0.63±0.13
**2**	EF-1α	V-ATP	V-ATP	QCR	EF-1α	QCR	EF-1α	EF-1α	EF-1α
**CV%±SD**	1.07±0.26	0.46±0.11	0.73±0.17	0.57±0.15	0.43±0.10	0.44±0.11	1.30±0.33	0.40±0.10	0.65±0.16
**3**	eIF-5A	CYP	EF-1α	EF-1α	CYP	ARF	pol IIa	SAR1	bTUB
**CV%±SD**	1.75±0.36	0.47±0.10	0.73±0.17	0.57±0.14	0.63±0.13	0.44±0.11	1.71±0.43	0.80±0.19	1.11±0.28
**4**	ARF	30SRPS20	ARF	SAR1	QCR	EF-1α	V-ATPase	eIF-5A	GAPDH
**CV%±SD**	1.90±0.45	0.61±0.15	1.12±0.27	1.17±0.29	0.79±0.20	0.75±0.19	1.78±0.41	0.84±0.17	1.45±0.32
**5**	SAR1	EF-1α	ACT1	18SrRNA	ACT1	V-ATP	aTUB	bTUB	ARF
**CV%±SD**	1.98±0.47	0.65±0.16	1.19±0.40	1.27±0.22	1.82±0.37	1.00±0.23	1.90±0.46	1.10±0.29	1.61±0.38
**6**	QCR	SAR1	6-PG	ARF	18SrRNA	CYP	eIF-5A	18SrRNA	QCR
**CV%±SD**	1.99±0.51	1.01±0.24	1.25±0.30	1.27±0.31	2.24±0.38	1.13±0.24	2.03±0.42	1.44±0.24	1.65±0.42
**7**	V-ATP	eIF-5A	GAPDH	30SRPS20	V-ATP	eIF-5A	bTUB	GAPDH	aTUB
**CV%±SD**	2.20±0.52	1.05±0.21	1.34±0.31	1.71±0.37	2.32±0.54	1.18±0.24	2.28±0.59	1.46±0.32	1.69±0.40
**8**	aTUB	6-PG	QCR	GAPDH	ARF	UBQ	QCR	aTUB	pol IIa
**CV%±SD**	2.36±0.56	1.17±0.27	1.4±0.36	1.86±0.41	2.43±0.57	1.77±0.39	2.62±0.67	1.82±0.45	1.74±0.45
**9**	GAPDH	F-box	60SRPL13	eIF-5A	eIF-5A	aTUB	ACT1	F-box	V-ATP
**CV%±SD**	2.39±0.53	1.24±0.28	1.92±0.48	2.08±0.44	2.50±0.52	1.87±0.45	2.91±0.62	1.99±0.45	2.09±0.49
**10**	ACT1	ARF	30SRPS20	CDP	SAR1	18SrRNA	6-PG	QCR	SAR1
**CV%±SD**	2.51±0.52	1.31±0.31	1.92±0.48	2.21±0.53	2.51±0.60	3.01±0.50	3.06±0.73	2.27±0.56	2.58±0.62

Notes: LP, leaf-expansion period; FS, the flower stage; GFS, the green fruit stage; RFS, the red fruit stages; RGS, the root growing after fruit stage. Descriptive statistics of 10 candidate genes based on the coefficient of variance (CV) and standard deviation (SD) of their Ct values were determined using the whole data set. Reference genes were identified as the most stable genes, i.e. those with the lowest coefficient of variance and standard deviation (CV% ± SD).

In summary, CYP and EF-1α were demonstrated to be the best reference genes under all the treatment conditions. In addition, GAPDH and V-ATP showed the highest CV±SD values (0.37±0.08 and 0.46±0.11, respectively) in the roots. However, ACT1 and QCR were the most stable reference genes in FS, and their CV±SD values were 0.29±0.06 and 0.44±0.11, respectively, which slightly differed between geNorm and NormFinder.

## Discussion

Selection of suitable reference genes is a crucial pre-condition to a successful gene expression study based on qRT-PCR. Using inaccurate reference genes can lead to conflicting results, particularly when the variations in the rate of transcription between sample groups are small [41]. Herein, we have described a systematic analysis involving the stability of mRNA expression of candidate genes for data normalization in qPCR experiments using different developmental stages and the three vegetative organs of *Panax ginseng*. Investigation of 20 candidate reference genes by Ct value, geNorm, NormFinder, and BestKeeper applets led to the identification of the best reference genes for differential gene expression analyses at different developmental stages and various organs of ginseng. In qRT-PCR analysis, certain housekeeping genes (such as, ACT, UBQ, F-box) are considered stably expressed in different environmental conditions and are commonly employed as reference gene(s) [36]. The analysis data revealed certain changes in the mRNA gene expression levels in majority of the traditional housekeeping genes of ginseng under different treatment conditions; therefore, these genes could not be considered as ideal ginseng reference genes. However, a stable reference gene is essential for genetic engineering studies in ginseng. To the best of our knowledge, this is the first report on the identification and validation of suitable reference genes for qRT-PCR analysis of ginseng.

An “ideal” reference gene(s) should be continually transcribed in all cell types and organs. Additionally, its RNA transcription level should be relatively constant in response to the internal and external stimulations [42]. For example, during housekeeping gene selection for qRT-PCR normalization in potato, it was found that the expression of EF-1α was not influenced by cold, salt, or late blight stressors [29]. In the analysis of reference genes for Arabidopsis, EF-1α was relatively stable in different organs [43]. However, under nutrition deficiency or abiotic stress, the stability of EF-1α was poor [44]. Selected as the appropriate reference gene in cucumber, the CYP gene was the most stable gene under cold and heat stress treatments; nevertheless it was less stable in various other tissues [45]. Based on our statistical analyses using Ct value, geNorm, NormFinder, and BestKeeper applets, the mRNA expression level of CYP, a traditional housekeeping gene, was found to be the most stable in different organs and developmental stages, and was followed by EF-1α (Table 5). Furthermore, out of the 10 novel reference genes, it was interesting to note that QCR was relatively stable in all the experimental samples.

**Table 5:** Stability ranking of 20 candidate reference genes using geNorm,Normfinder and Bestkeeper.

	Total			Root			Stem			Leaf			LP			FS			GFS			RFS			RGS		
	G	N	B	G	N	B	G	N	B	G	N	B	G	N	B	G	N	B	G	N	B	G	N	B	G	N	B
**CYP**	**1**	**1**	**1**	5	4	3	**1**	**8**	**1**	**1**	**1**	**1**	**1**	**1**	**3**	**1**	**2**	**6**	9	9	1	4	9	1	7	11	1
**EF-1α**	**1**	**4**	**2**	9	7	5	8	3	3	4	4	3	3	7	2	3	6	4	4	11	2	5	11	2	8	12	2
**QCR**	4	2	6	15	15	—*	6	1	7	**1**	**2**	**2**	4	2	4	4	8	2	6	8	8	12	8	10	5	1	6
**eIF-5A**	3	3	3	4	8	7	14	13	—	10	11	9	10	4	9	**1**	**5**	**7**	3	5	6	**1**	**5**	**4**	18	18	—
**ACT1**	7	5	10	16	14	—	9	9	8	12	10	—	6	10	5	6	9	1	7	10	9	13	10	—	14	14	—
**V-ATP**	8	6	7	3	2	2	7	10	2	17	19	—	11	5	7	5	4	5	**1**	**13**	**4**	14	13	—	6	2	9
**GAPDH**	11	7	9	**1**	**1**	**1**	4	5	6	5	5	8	17	14	—	13	13	—	17	7	—	9	7	7	3	6	4
**ARF**	5	8	4	6	9	10	5	2	4	9	6	6	9	6	8	7	10	3	11	15	—	15	15	—	9	8	5
**UBQ**	12	9	—	8	13	—	13	11	—	20	20	—	14	15	—	8	3	8	15	14	—	10	14	—	10	10	—
**pol IIa**	14	10	—	14	16	—	12	12	—	13	15	—	13	12	—	14	11	—	5	16	3	17	16	—	**1**	**4**	**8**
**SAR1**	6	11	5	12	6	6	11	14	—	7	7	4	8	8	10	12	17	—	13	6	—	**1**	**6**	**3**	11	7	10
**aTUB**	9	12	8	10	12	—	16	13	—	14	14	—	7	11	—	9	1	9	**1**	**1**	**5**	7	1	8	**1**	**3**	**7**
**bTUB**	10	13	—	11	11	—	17	19	—	16	16	—	15	18	—	11	12	—	16	4	7	6	4	5	4	5	3
**F-box**	15	14	—	7	10	9	15	15	—	19	7	—	15	17	—	16	15	—	10	2	—	8	2	9	15	15	—
**18SrRNA**	13	15	—	20	20	—	18	18	—	3	3	5	5	9	6	10	7	10	14	3	—	3	3	6	16	16	—
**60SRPL13**	17	16	—	19	19	—	**1**	**7**	**9**	18	18	—	**1**	**3**	**1**	17	16	—	18	12	—	11	19	—	12	9	—
**6-PG**	16	17	—	13	5	8	3	6	5	11	13	—	12	13	—	15	14	—	8	17	10	16	12	—	19	19	—
**CDP**	19	18	—	17	17	—	19	17	—	8	8	10	16	16	—	18	18	—	19	18	—	19	17	—	13	13	—
**TCTP**	18	19	—	18	18	—	20	20	—	15	12	—	19	19	—	19	19	—	12	19	—	18	18	—	17	17	—
**30SRPS20**	20	20	—	**1**	**3**	**4**	10	4	10	6	9	7	20	20	—	20	20	—	20	20	—	20	20	—	20	20	—

Notes: LP, leaf-expansion period; FS, the flower stage; GFS, the green fruit stage; RFS, the red fruit stages; RGS, the root growing after fruit stage. G,geNorm software; N, Normfinder software; B, Bestkeeper software. * means It has not been testing by Bestkeeper.

Although the results of all the three applets were reasonable, they were not found to be completely consistent. However, this variation was not surprising, since the three software applications are based on different calculation algorithms [25]. geNorm is known to be a more effective and feasible algorithm for ensuring the optimal stability of reference genes, whereas NormFinder and BestKeeper are best applied for assessing the quality of the gene rankings obtained by geNorm [26], [46], [47]. The results of the geNorm analysis have been satisfactorily accepted by many researchers [21]-[26], [48], [49]. In the present study, the two top ranked reference genes for the total samples, roots, leaves, and the developmental stage, LP, obtained through geNorm were consistent with the ranking of NormFinder and BestKeeper. However, the two best ranked reference genes in the stems and other developmental stages (FS, GFS, RFS, and RGS), as analyzed by geNorm, were slightly different from the results produced by NormFinder or BestKeeper; interestingly, the genes were still top-ranked. Our data showed that CYP and EF-1α were the most stable reference genes among all the samples. Meanwhile, different types of samples revealed their own best reference genes amongst the 20 selected candidate reference genes. In the different vegetative organs of ginseng, GAPDH and 30SRPS20 were the best reference genes found in the roots; CYP and 60SRPL13 were the top-ranked reference genes in the stems; and CYP and QCR were the best reference genes in the leaves. In different developmental stages of ginseng, CYP/60SRPL13, CYP/eIF-5A, aTUB/V-ATP, eIF-5A/SAR1, and aTUB/pol IIa were the most stably expressed combinations in LP, FS, GFS, RFS, and RGS, respectively. Their CV and MFC values were relatively low. Although 30SRPS20 was the least stable among the 20 candidate reference genes in all five developmental stages, it ranked high in the roots, as determined by geNorm, NormFinder, and BestKeeper.

Taken together, we identified 20 potential reference genes from 15 *P. ginseng* samples (different organs and developmental stages) for the normalization of qRT-PCR data. CYP and EF-1α were the most suitable reference genes in ginseng, as evaluated by the three software applications.

## Conclusion

Gene transcription studies using real-time quantitative reverse transcription-polymerase chain reaction (qRT-PCR) necessitate the selection of appropriate reference genes that are reliable under various experimental conditions. Consistent with other reports in the literature [50], we agree that more than one gene should be used as reference genes to obtain reliable results in gene transcription analyses. This study systematically expounds a new way to screen for candidate reference genes on the basis of the Illumina sequencing platform, and subsequently identifies a set of the most stable reference genes in different vegetative organs and different developmental stages of *P. ginseng*. The present study will therefore provide greater accuracy and normalization to qRT-PCR analysis in future ginseng research.
